# 

**Published:** 1893-10

**Authors:** 


OCTOBER, 1893.

### THE AMERICAN JOURNAL OF DENTAL SCIENCE.

PUBLISHED MONTHLY.

$2.50 PER ANNUM.

EDITED BY

F. J. S. GORGAS, M. D., D. D. S.
RICHARD GRADY, M. D., D. D. S.

Vol. 27. THIRD SERIES No. 6.

BALTIMORE:
SNOWDEN & COWMAN, 9 W. Fayette Street.

LONDON:
TRUBNER & CO., 60 Paternoster Row

Entered at the Post Office at Baltimore, Md., as Second-class Matter.

PAYABLE IN ADVANCE.

